# Analysis of the Complete Genome of the Alkaliphilic and Phototrophic Firmicute *Heliorestis convoluta* Strain HH^T^

**DOI:** 10.3390/microorganisms8030313

**Published:** 2020-02-25

**Authors:** Emma D. Dewey, Lynn M. Stokes, Brad M. Burchell, Kathryn N. Shaffer, Austin M. Huntington, Jennifer M. Baker, Suvarna Nadendla, Michelle G. Giglio, Kelly S. Bender, Jeffrey W. Touchman, Robert E. Blankenship, Michael T. Madigan, W. Matthew Sattley

**Affiliations:** 1 Division of Natural Sciences, Indiana Wesleyan University, Marion, IN 46953, USA; emma.dewey@myemail.indwes.edu (E.D.D.); lynn.stokes@myemail.indwes.edu (L.M.S.); brad.burchell@indwes.edu (B.M.B.); kshaffer@cedarville.edu (K.N.S.); austinm.huntington@gmail.com (A.M.H.); jennbak@umich.edu (J.M.B.); 2 Institute for Genome Sciences, University of Maryland School of Medicine, Baltimore, MD 21201, USA; snadendla@som.umaryland.edu (S.N.); mgiglio@som.umaryland.edu (M.G.G.); 3 Department of Microbiology, Southern Illinois University, Carbondale, IL 62901, USA; bender@micro.siu.edu (K.S.B.); madigan@siu.edu (M.T.M.); 4 School of Life Sciences, Arizona State University, Tempe, AZ 85287, USA; jefftouchman@icloud.com; 5 Departments of Biology and Chemistry, Washington University in Saint Louis, St. Louis, MO 63130, USA; blankenship@wustl.edu

**Keywords:** heliobacteria, *Heliorestis convoluta*, alkaliphilic bacteria, soda lake, anoxygenic phototroph, bacteriochlorophyll *g*

## Abstract

Despite significant interest and past work to elucidate the phylogeny and photochemistry of species of the *Heliobacteriaceae*, genomic analyses of heliobacteria to date have been limited to just one published genome, that of the thermophilic species *Heliobacterium* (*Hbt*.) *modesticaldum* str. Ice1^T^. Here we present an analysis of the complete genome of a second heliobacterium, *Heliorestis* (*Hrs.*) *convoluta* str. HH^T^, an alkaliphilic, mesophilic, and morphologically distinct heliobacterium isolated from an Egyptian soda lake. The genome of *Hrs. convoluta* is a single circular chromosome of 3.22 Mb with a GC content of 43.1% and 3263 protein-encoding genes. In addition to culture-based observations and insights gleaned from the *Hbt. modesticaldum* genome, an analysis of enzyme-encoding genes from key metabolic pathways supports an obligately photoheterotrophic lifestyle for *Hrs. convoluta*. A complete set of genes encoding enzymes for propionate and butyrate catabolism and the absence of a gene encoding lactate dehydrogenase distinguishes the carbon metabolism of *Hrs. convoluta* from its close relatives. Comparative analyses of key proteins in *Hrs. convoluta*, including cytochrome *c*_553_ and the F_o_ alpha subunit of ATP synthase, with those of related species reveal variations in specific amino acid residues that likely contribute to the success of *Hrs. convoluta* in its highly alkaline environment.

## 1. Introduction

Heliobacteria comprise a unique group of strictly anaerobic, anoxygenic phototrophs that have been isolated from a wide diversity of soil and aquatic habitats [1,2,3,4]. Unlike all other phototrophic bacteria, heliobacteria use bacteriochlorophyll (Bchl) *g* as the chief chlorophyll pigment for phototrophic growth [5], but despite their ability to use light as an energy source, heliobacteria are apparently incapable of autotrophic growth and, thus, are obligate heterotrophs [4,6]. Heliobacteria are the only phototrophs of the large bacterial phylum *Firmicutes* [4,7,8], and although they typically stain Gram-negatively, thin sections of cells of heliobacteria exhibit a Gram-positive cell wall morphology [9,10]. In addition to these distinctive properties, cells of heliobacteria are able to differentiate into heat-resistant endospores [4,11], and some heliobacteria have also demonstrated the ability to reduce toxic metals, such as Hg^2+^, and therefore may be useful for applications in bioremediation [12,13].

**Figure 1:**
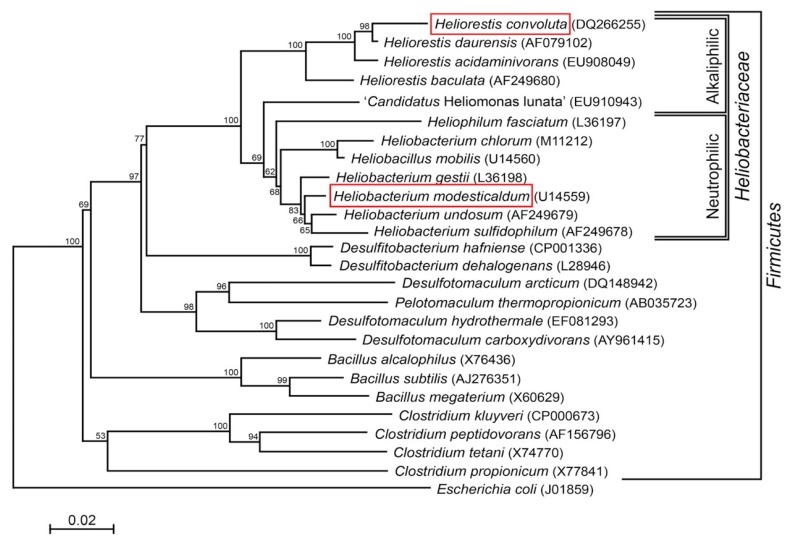
Phylogenetic (16S rRNA) tree of *Heliorestis convoluta* and related *Firmicutes*. Heliobacteria, the only phototrophic *Firmicutes*, are divided into alkaliphilic and neutrophilic species. *Heliobacterium modesticaldum* (boxed) is the model organism for physiological and biochemical studies of the heliobacteria; *Hrs. convoluta* (boxed) is the first alkaliphilic heliobacterium to have a described genome. Note that the branching pattern shown here suggests a possible alkaliphilic origin to the heliobacteria, as previously discussed by Sattley and Swingley [7]. The weighted neighbor-joining method [18] and Jukes-Cantor corrected distance model were used for tree construction. Nodes represent bootstrap values (≥50%) based on 100 replicates, and *Escherichia coli* was used to root the tree. GenBank accession numbers for each sequence used in the analysis are shown in parentheses, adapted from Sattley and Swingley [7], *Adv. Bot. Res.* **2013**, *66*, 67–97, Copyright 2013 Elsevier Ltd.

Species of *Heliobacteriaceae* can be divided into two physiological groups—neutrophiles and alkaliphiles—that track closely with their phylogeny [7] (Figure 1). Included in the neutrophilic clade, the moderate thermophile *Hbt. modesticaldum* was the first heliobacterium to have its genome sequenced and, with its simple phototrophic machinery consisting of a type I reaction center (RC) and no peripheral antenna photocomplex, has been a model organism for studies of photosynthesis and related photochemistry [6,14]. Like other neutrophilic heliobacteria, *Hbt. modesticaldum* exhibits both phototrophic growth in the light and chemotrophic growth in the dark [3,15,16,17]. 

**Figure 2:**
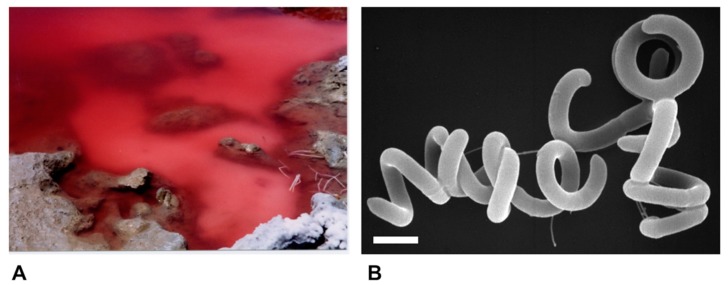
Habitat and cells of *Heliorestis convoluta* strain HH^T^. (**A**) Red bloom of alkaliphilic *Bacteria* and *Archaea* on the shore of Lake El Hamra, Wadi Natroun, Egypt. M.T.M. sampled this bloom in May 2001, and enrichments for heliobacteria yielded *Hrs. convoluta*. The bloom is about 2 m in diameter.; (**B**) Scanning electron micrograph of cells of *Hrs. convoluta* strain HH^T^. A cell of *Hrs. convoluta* is about 0.5 μm in diameter and coils are of variable length. Scale bar = 1 μm.

Species of alkaliphilic heliobacteria grow optimally between pH 8–9.5 and, unlike neutrophilic heliobacteria, are obligate photoheterotrophs, using light and organic compounds for growth but incapable of chemotrophic growth in darkness [19,20,21,22]. Consistent with other alkaliphilic heliobacteria originating from the soils and waters of soda lakes [19,20,22], *Hrs. convoluta* str. HH^T^ was isolated from the shore of the alkaline (pH 10) Lake El Hamra (Figure 2A), located in the Wadi El Natroun region of northern Egypt [21]. In the past, the saline lakes of the Wadi El Natroun have also been a fertile source of alkaliphilic purple bacteria, yielding many extremely alkaliphilic (and in some cases also extremely halophilic) species, including in particular, new species of the genus *Halorhodospira* [23,24,25]. However, *Hrs. convoluta* is the first heliobacterium to originate from these unusual lakes. Experimental work with *Hrs. convoluta* revealed motile cells having an unusual tightly coiled morphology (Figure 2B) and displaying a mesophilic (optimal growth at 33 °C) and alkaliphilic (optimal growth at pH 8.5–9) physiology [21].

To complement the analysis of the genome sequence of *Hbt. modesticaldum* [6,26], we present here a comparative analysis of the genome of *Hrs. convoluta*. Although a number of highly conserved genes encoding proteins that coordinate key processes in the cell (e.g., phototrophy and central carbon metabolism) are shared between these species, a close comparison of the two heliobacterial genomes revealed several genes encoding functions in carbon metabolism, biotin biosynthesis, nitrogen and sulfur assimilation, and carotenoid biosynthesis that are not held in common by these heliobacteria, which inhabit vastly different extreme environments. In addition, a comparative analysis of selected cytochrome and ATP synthase proteins in *Hrs. convoluta* revealed adaptations that likely facilitate its alkaliphilic lifestyle. The availability of a second heliobacterial genome, as well as the recent development of a genetic system in *Hbt. modesticaldum* [14], paves the way for increasing our understanding of the unique metabolism and physiology of heliobacteria.

## 2. Materials and Methods

Total genomic DNA from *Hrs. convoluta* str. HH^T^ (ATCC BAA-1281 and DSMZ 19787) [21] was isolated through proteinase K treatment and subsequent phenol extraction. Complete genome sequencing was performed using a random shotgun approach, and reads were assembled using Velvet v. 2010 [27]. Pyrosequencing on a Roche-454 GS20 sequencer (Hoffman-La Roche AG, Basel, Switzerland) provided 14-fold genome coverage, and an additional 35-fold coverage was generated by the Illumina GAIIx platform.

Annotation of the *Hrs. convoluta* genome was performed in accordance with the Prokaryotic Annotation Pipeline of the University of Maryland School of Medicine’s Institute for Genome Sciences [28]. This pipeline employs Glimmer for gene identification and then searches the protein sequences with BLAST-extend-repraze (BER; a combination of BLAST and Smith–Waterman algorithms) to generate pairwise alignments, Hidden Markov Model (HMM), transmembrane (Tm) HMM, and SignalP predictions. An automated process employing the Pfunc evidence hierarchy is used to assign functional annotations. Manual verification of automated annotations was facilitated through the online tool Manatee [29] in conjunction with online databases including the Kyoto Encyclopedia of Genes and Genomes (KEGG), the Braunschweig Enzyme Database (BRENDA), MetaCyc, and Uniprot. The National Center for Biotechnology Information (NCBI) database was accessed to retrieve gene and protein sequences from related species for comparative analyses with corresponding genes in the genome of *Hrs. convoluta*. 

The phylogenetic tree was generated as described in the legend to Figure 1. Genome statistics were compiled using the Pfam database v. 30.0 [30], the SignalP database v. 4.1 [31], the TMHMM database v. 2.0 [32], and CRISPRFinder v. 2.0 [33]. This complete genome sequence project has been deposited at DDBJ/EMBL/GenBank under accession number CP045875.

## 3. Results and Discussion

### 3.1. Genome Properties

The 3,218,981 base-pair (bp) genome of *Heliorestis convoluta* str. HH^T^ is organized into a single circular chromosome with no plasmids (Table 1). The 43.1% GC content of *Hrs. convoluta* is among the lowest of all heliobacteria (41%–57.7%) and is typical of alkaliphilic species of this group of phototrophs [4]. Nearly 87% of the *Hrs. convoluta* genome content is protein-encoding, with a total of 3263 protein coding genes at an average length of 855 nucleotides (Table 1). The genome contains nine ribosomal RNA (rRNA) genes, including multiple copies each of 5S, 16S (two full and one partial), and 23S rRNA, which are distributed randomly on the chromosome. Nearly 11% of the open reading frames (ORFs) were of unknown enzyme specificity or function, and 28% of genes were annotated as hypothetical. The role category breakdown of protein-encoding genes of *Hrs. convoluta* is shown in Table 2. 

Genes encoding a total of 105 transfer RNAs (tRNAs) were identified in the *Hrs. convoluta* genome, as well as genes encoding all twenty common aminoacyl-tRNA synthetases except asparaginyl-tRNA synthetase, which could not be confirmed. However, genes encoding aspartyl/glutamyl-tRNA amidotransferase (*gatABC*) were identified in *Hrs. convoluta* and, as proposed for *Hbt. modesticaldum* [6], may encode a protein that compensates for the missing asparaginyl-tRNA synthetase by converting aspartyl-tRNA to asparaginyl-tRNA [34,35].

### 3.2. Central Carbon Metabolism

**Figure 3:**
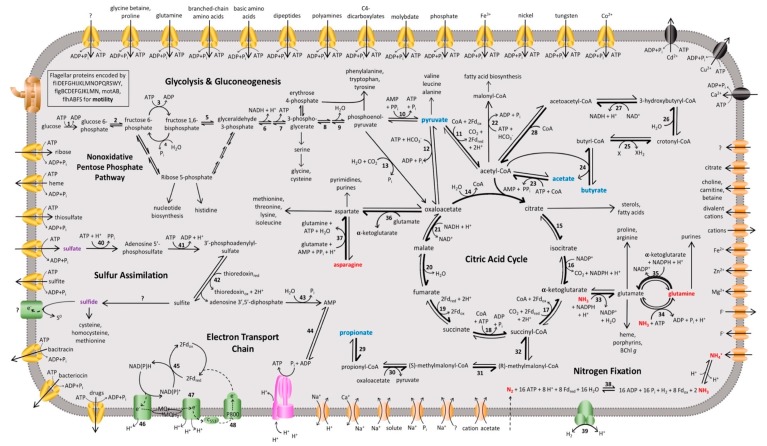
Overview of the proposed metabolic pathways and membrane transporters in *Heliorestis convoluta*. Carbon (blue), nitrogen (red), and sulfur (purple) sources are catabolized or assimilated for phototrophic growth of *Hrs. convoluta*. The predicted dominant direction of metabolic flow is shown by bolded arrows. Numbers signify enzymes identified in the genome of *Hrs. convoluta*, whereas question marks indicate unidentified but anticipated enzymes catalyzing the respective reaction. Enzymes involved in glycolysis or gluconeogenesis include (1) glucokinase, (2) glucose-6-phosphate isomerase, (3) 6-phosphofructokinase, (4) fructose 1,6-bisphosphatase, (5) fructose-1,6-bisphosphate aldolase, (6) glyceraldehyde-3-phosphate dehydrogenase, (7) phosphoglycerate kinase, (8) phosphoglycerate mutase, (9) enolase, and (10) pyruvate-phosphate dikinase. CAC enzymes are (11) pyruvate:ferredoxin oxidoreductase, (12) pyruvate carboxylase, (13) phosphoenolpyruvate carboxylase, (14) citrate (*re*)-synthase, (15) aconitate hydratase, (16) NADP^+^-dependent isocitrate dehydrogenase, (17) 2-oxoglutarate synthase/2-oxoglutarate:ferredoxin oxidoreductase, (18) succinyl-CoA synthetase, (19) succinate dehydrogenase/fumarate reductase, (20) fumarate hydratase, and (21) NAD^+^-dependent malate dehydrogenase. Acetyl-CoA metabolism is carried out by (22) acetyl-CoA carboxylase and (23) acetyl-CoA synthetase. Butyrate metabolism enzymes include (24) CoA transferase, (25) acyl-CoA dehydrogenase, (26) enoyl-CoA hydratase, (27) 3-hydroxybutyryl-CoA dehydrogenase, and (28) acetyl-CoA C-acetyltransferase. Propionate metabolism is catabolized by (29) CoA transferase, (30) methylmalonyl-CoA carboxytransferase, (31) methylmalonyl-CoA epimerase, and (32) methylmalonyl-CoA mutase. Amino acid metabolism enzymes are (33) NADP^+^-specific glutamate dehydrogenase, (34) glutamine synthetase, (35) NADPH-dependent glutamate synthase, (36) pyridoxal phosphate-dependent aminotransferase, and (37) asparagine synthase. The enzymes (38) nitrogenase and (39) uptake [NiFe] hydrogenase catalyze nitrogen fixation and H_2_ oxidation, respectively, and sulfur assimilation is performed by (40) sulfate adenyltransferase, (41) adenylyl-sulfate kinase, (42) phosophoadenylyl-sulfate reductase, (43) bifunctional oligoribonuclease and PAP phosphatase, and (44) adenylate kinase. Finally, the electron transport chain includes (45) ferredoxin:NADP^+^ reductase, (46) NADH:quinone oxidoreductase, (47) cytochrome *bc* complex, and the (48) light-harvesting reaction center. Membrane proteins include ABC transporters (yellow), P-type ATPases (black), ATP synthase (pink), flagellar and motor proteins (brown), other transporters (orange), and other membrane proteins (green).

Analysis of the *Hrs. convoluta* genome confirmed culture-based observations of the limited set of carbon sources able to support light-driven growth of this species [21]. As an obligate photoheterotroph, *Hrs. convoluta* grows only in anoxic, light conditions when supplied with mineral media containing CO_2_ plus acetate, pyruvate, propionate, or butyrate as organic carbon sources [21]. Of the 12 described species of heliobacteria (Figure 1), only *Heliorestis acidaminivorans*, *Heliorestis daurensis*, and *Hrs. convoluta* are capable of propionate photoassimilation [4,19,21,22]. Genes encoding enzymes of the methylmalonyl pathway, which converts propionyl-coenzyme A (CoA) to succinyl-CoA for propionate assimilation, were identified in the *Hrs. convoluta* genome (Figure 3). Although a gene encoding propionyl-CoA carboxylase, which is thought to catalyze the first step in the proposed pathway [36,37], was not identified in the *Hrs. convoluta* genome, a gene predicted to encode methylmalonyl-CoA carboxyltransferase (FTV88_3237), which could circumvent this deficiency, was identified. 

Unlike other *Heliorestis* species, *Hrs. convoluta* and a few other heliobacteria can use butyrate as a carbon source [19,20,21,38]. Analysis of the *Hrs. convoluta* genome revealed genes encoding enzymes that catabolize butyrate to acetyl-CoA for incorporation into the citric acid cycle (CAC) [39] (Figure 3). Genes encoding butyryl-CoA:acetate CoA transferase, which catalyzes the conversion of butyrate to butyryl-CoA in butyrate catabolism [39], and propionyl-CoA synthetase, which converts propionate to propionyl-CoA in propionate catabolism [37], were not identified in the genome of *Hrs. convoluta*. However, an experimentally characterized butyryl-CoA:acetate CoA transferase from *Desulfosarcina cetonica* [39] showed 47% amino acid sequence identity with 4-hydroxybutyrate CoA-transferase (FTV88_0224) from *Hrs. convoluta.* In addition, the product of a gene annotated as acetyl-coenzyme A synthetase (FTV88_0994) in *Hrs. convoluta* showed 37% sequence identity with propionyl-CoA synthetase from *Salmonella enterica* and contained the conserved lysine residue (Lys592) required in the initial reaction of propionate catabolism [40]. These findings suggest possible roles for 4-hydroxybutyrate CoA-transferase and acetyl-coenzyme A synthetase in butyrate and propionate catabolism, respectively, in *Hrs. convoluta*.

Although capable of growth on pyruvate, *Hrs. convoluta* str. HH^T^ is unable to grow photoheterotrophically on lactate [21], a phenotype distinct from that of most other heliobacteria and the result of an underlying genetic deficiency. In this connection, a gene encoding a putative L-lactate dehydrogenase in *Hbt. modesticaldum* [6] showed no meaningful similarity to any genes in *Hrs. convoluta*. In addition to lactate, no growth was detected when alcohols of any kind were used as sole carbon source in cultures of strain HH^T^ [21]. Despite this observation, genes encoding alcohol dehydrogenase and aldehyde dehydrogenase were annotated in the *Hrs. convoluta* genome and, thus, could potentially play a role in non-energetic processes, such as detoxification.

Although a full complement of genes encoding enzymes of the glycolytic and nonoxidative pentose phosphate pathways was present in the *Hrs. convoluta* genome (Figure 3), various common sugars did not support photoheterotrophic growth of strain HH^T^ [21]. An inability to use sugars was also originally reported for *Hbt. modesticaldum* [16], but later experimentation showed that *Hbt. modesticaldum* utilized the glycolytic pathway when D-ribose, D-glucose, or D-fructose were supplied with low levels of yeast extract [41]. Although no gene encoding a hexose transporter was annotated in the *Hrs. convoluta* genome, a putative ribose ABC transporter complex (FTV88_0053, FTV88_0054, FTV88_0055) was identified and may allow for carbohydrate transport [41]. As genes encoding glycolytic pathway enzymes are present in the *Hrs. convoluta* genome, it is tempting to speculate that the alkaliphile can utilize sugars in a manner similar to *Hbt. modesticaldum*. The absence of genes encoding glucose 6-phosphate dehydrogenase and 6-phosphogluconolactonase suggest incomplete Entner-Doudoroff and oxidative pentose phosphate pathways, which was also the case for *Hbt. modesticaldum* [6].

It is likely that *Hrs. convoluta* can catalyze many of the steps in the CAC based on biochemical studies of *Hbt. modesticaldum* [42] and high sequence similarity of key CAC enzymes between the two species (Figure 3). However, since both *Hrs. convoluta* and *Hbt. modesticaldum* lack a gene encoding pyruvate dehydrogenase for oxidizing pyruvate to acetyl-CoA, this reaction in heliobacteria is likely catalyzed by the enzyme pyruvate:ferredoxin oxidoreductase (PFOR); the gene encoding PFOR in *Hrs. convoluta* (FTV88_3370) shares 61% sequence identity to an orthologous gene in *Hbt. modesticaldum* [6]. Furthermore, an unusual citrate synthase, citrate (*re*)-synthase, which specifically catalyzes the addition of the acetyl moiety from acetyl-CoA to the *re* face of the ketone carbon of oxaloacetate [a stereospecificity opposite to that of citrate (*si*)-synthase], has been identified in several clostridia and other strictly anaerobic *Firmicutes*, including *Hbt. modesticaldum* [42]. In *Hrs. convoluta*, a gene (FTV88_1447) having high amino acid sequence identity (81%) to the gene encoding citrate (*re*)-synthase (HM1_2993) in *Hbt. modesticaldum* supports the presence of citrate (*re*)-synthase in *Hrs. convoluta* and suggests this unusual form of citrate synthase is common to all heliobacteria.

In regards to photoautotrophic capacity, no genes encoding enzymes of any form of the Calvin-Benson cycle, including ribulose 1,5-bisphosphate carboxylase and phosphoribulokinase, were identified in the *Hrs. convoluta* genome. In addition, the lack of genes encoding key enzymes of other autotrophic pathways, such as malyl-CoA lyase (3-hydroxypropionate/4-hydroxybutyrate pathway) and acetyl-CoA synthase (Wood-Ljungdahl pathway), also prevents *Hrs. convoluta* from assimilating CO_2_ into organic carbon molecules for growth. The capacity for CO_2_ fixation by the reverse CAC, as observed in green sulfur bacteria [43], is apparently disrupted by the absence of a gene encoding ATP-citrate lyase. Although an ORF identified as a citrate lyase family protein (FTV88_0308) was annotated in the *Hrs. convoluta* genome based on sequence identities of approximately 50% with corresponding genes from other *Firmicutes* (but having no similarity to genes in *Hbt. modesticaldum*), biochemical analysis of this gene product would be required to assess its activity and role, if any, in metabolic pathways of *Hrs. convoluta*. Although anapleurotic CO_2_ assimilation has been shown in heliobacteria supplied with usable organic carbon sources [44], cultures of *Hrs. convoluta* strain HH^T^, like all other cultured heliobacteria, were unable to grow using CO_2_ as sole carbon source [21], thus supporting the premise that heliobacteria require an organic carbon source during phototrophic growth. 

In addition to phototrophy, neutrophilic heliobacteria are able to grow chemotrophically in the dark by pyruvate fermentation [4]. Interestingly, however, the capacity for pyruvate fermentation has not been observed in any alkaliphilic heliobacterial isolate to date, including *Hrs. convoluta* [4,15,17,21]. Studies have suggested that the neutrophile *Hbt. modesticaldum* carries out substrate-level phosphorylation via acetyl-CoA conversion to acetate in dark, anoxic (fermentative) conditions through the activity of phosphotransacetylase (PTA) and acetate kinase (ACK) [15,17,41]. A gene encoding ACK (FTV88_2009) was annotated in the genome of *Hrs. convoluta* and has 67% sequence identity to a corresponding gene in *Hbt. modesticaldum*. However, a gene encoding PTA could not be identified in either *Hrs. convoluta* or *Hbt. modesticaldum*. Therefore, the genetic determinants that coordinate pyruvate fermentation in neutrophilic heliobacteria but are apparently absent from alkaliphilic heliobacteria remain unidentified.

Three *Hrs. convoluta* genes encoding acetyl-CoA synthetase (ACS) were identified in the genome, one of which showed 87% amino acid sequence identity with the corresponding gene in *Hbt. modesticaldum*. Activity of ACS in *Hbt. modesticaldum* cell extracts was detected only under phototrophic (light/anoxic) conditions, and expression levels of the ACS gene decreased when the bacterium was cultured in darkness [41], thus indicating that, although technically reversible, ACS activity is predominately skewed toward the production of acetyl-CoA from acetate (Figure 3). Activity of ACS therefore allows both *Hbt. modesticaldum* and *Hrs. convoluta* to grow photoheterotrophically using acetate as sole carbon source [16,21]. 

In contrast to all other heliobacteria, which require biotin for growth, *Hrs. convoluta* and close relative *Hrs. acidaminivorans* (Figure 1) have no growth factor requirements [21,22]. The presence of a full complement of genes (*bioABCDF*) encoding enzymes for biotin biosynthesis allows *Hrs. convoluta* to synthesize biotin, thereby supporting culture-based observations [21]. By contrast, analysis of the *Hbt. modesticaldum* genome revealed the absence of two key genes for biotin biosynthesis, *bioC* and *bioF*, thus explaining the absolute requirement for biotin in that species [16].

### 3.3. Nitrogen Metabolism

*Hrs. convoluta* is strongly diazotrophic [21], and as in *Hbt. modesticaldum*, genes for nitrogen fixation are grouped into a single *nif* gene cluster containing *nifI_1_*, *nifI_2_*, *nifH*, *nifD*, *nifK*, *nifE*, *nifN*, *nifX*, *fdxB*, *nifB*, and *nifV* [6]. Each of these genes shows between 63% and 93% sequence identity and analogous gene synteny to corresponding genes in *Hbt. modesticaldum*. A study with *Paenibacillus* sp. WLY78—also an endospore-former within the phylum *Firmicutes*—concluded that nine genes (*nifB*, *nifH*, *nifD*, *nifK*, *nifE*, *nifN, nifX*, *hesA*, *nifV*), which were grouped into a single gene cluster, are essential to synthesize a catalytically-active nitrogenase for dinitrogen assimilation [45]. All of these nitrogen fixation genes, except for *hesA*, were identified in the *Hrs. convoluta* and *Hbt. modesticaldum* genomes. Since HesA is proposed to play a role in metallocluster biosynthesis [45], it is possible that a gene (FTV88_2056) located outside of the *nif* gene cluster and encoding a putative dinitrogenase Fe/Mo cofactor biosynthesis protein fills this role in *Hrs. convoluta*. This encoded protein showed high (~64%) sequence identity to a corresponding protein in *Hrs. acidaminivorans* and over 50% sequence similarity to that from a variety of nonphototrophic *Firmicutes*, but it showed no significant similarity to proteins encoded by *Hbt. modesticaldum*. 

Research on *Hbt. modesticaldum* revealed changes in expression levels of numerous genes essential for various metabolic, biosynthetic, and other cellular pathways when the organism was grown under N_2_-fixing conditions [46]. This diazotrophic effect likely exists in other heliobacteria as well, including *Hrs. convoluta*. In terms of regulation of nitrogen fixation genes, however, it is interesting that neither *orf1* nor *nifA*, which encode regulatory proteins for the expression of *nif* structural genes [47,48], could be identified in the *Hrs. convoluta* genome. In *Hbt. modesticaldum*, the *orf1* gene product likely regulates the expression of *nif* genes when levels of fixed nitrogen are too low to support non-diazotrophic growth of the organism [16,48]. It is possible that *Hrs. convoluta* lacks the *orf1* and *nifA* regulatory genes and instead employs only *nifI_1_* (FTV88_2453) and *nifI_2_* (FTV88_2454) to coordinate post-translational regulation of nitrogenase [49]. 

**Figure 4:**
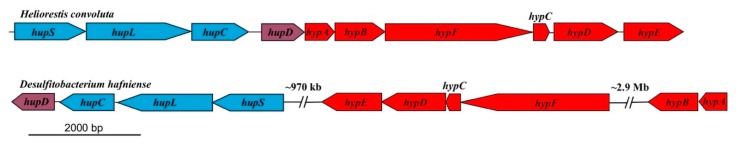
Comparison of uptake [NiFe]-hydrogenase genes in related *Firmicutes*. The genes are concatenated within a single region in *Heliorestis convoluta*, but they are dispersed in different regions of the *Desulfitobacterium hafniense* chromosome. Colors: blue, [NiFe]-hydrogenase structural genes; purple, hydrogenase expression/formation; red, hydrogenase assembly/maturation. Adapted from Sattley et al. [6]. *J. Bacteriol.* **2008**, *190*, 4687–4696. Copyright 2008 American Society for Microbiology.

*Hrs. convoluta* and *Hbt. modesticaldum* both contain gene clusters (*hypABCDEF* and *hupCDLS*) that encode an uptake [NiFe] hydrogenase that can putatively catalyze the oxidation of H_2_ produced during nitrogen fixation [6] (Figure 3). The arrangement of these genes in *Hrs. convoluta* is identical to that reported for *Hbt. modesticaldum* [6], being organized into a single cluster instead of dispersed throughout different regions of the chromosome, as has been observed in the genomes of other *Firmicutes* (Figure 4).

In addition to performing N_2_ fixation, cells of *Hrs. convoluta* strain HH^T^ could assimilate ammonia, glutamine, and asparagine as nitrogen sources [21]. Accordingly, genes encoding the ammonium transporter protein Amt (FTV88_2595) and enzymes of the glutamine synthetase-glutamate synthase pathway, which incorporates ammonia in the formation of glutamine from glutamate [50,51] (Figure 3), were identified in the *Hrs. convoluta* genome. Following transport, glutamine can then be used for purine biosynthesis or, through the activity of NADPH-dependent glutamate synthase, can be condensed with α-ketoglutarate to yield two molecules of glutamate for other biosynthetic pathways [50,51]. In addition, a gene encoding NADP-specific glutamate dehydrogenase (FTV88_2506) enables *Hrs. convoluta* to assimilate ammonia when synthesizing glutamate directly from α-ketoglutarate (Figure 3). Finally, genes encoding a glutamine-hydrolyzing asparagine synthetase (FTV88_1161 and FTV88_3319), which converts asparagine and glutamate into aspartate and glutamine, respectively (Figure 3), allow for the use of asparagine as a nitrogen source. In contrast, aspartate and glutamate cannot serve as nitrogen sources for strain HH^T^ [21]. Taken together, these findings suggest that, although the reactions are generally considered reversible, the enzymes catalyzing the conversion of asparagine to aspartate and glutamine to glutamate are physiologically unidirectional, strongly favoring the formation of aspartate and glutamate, respectively (shown as bolded arrows in Figure 3).

### 3.4. Assimilation of Sulfur

Growth studies indicate *Hrs. convoluta* is capable of assimilatory sulfate reduction [21]. Consistent with these observations, genomic analyses revealed that the pathway of assimilatory sulfate reduction in *Hrs. convoluta* begins with sulfate uptake using a sulfate/thiosulfate ABC transporter (*cysAWTP*). Typically, an enzyme encoded by *cysD* and *cysN*, sulfate adenyltransferase, catalyzes the assimilation of sulfate as adenosine phosphosulfate (APS) [52,53]. *Hrs. convoluta* lacks *cysD*, but genes encoding the bifunctional enzyme CysN/CysC (FTV88_1460 and FTV88_1458, respectively), which can also perform this function [54], are present. As shown in Figure 3, adenylyl-sulfate kinase (*cysC*) and phosphoadenylyl-sulfate reductase (*cysH*, FTV88_1461) catalyze the subsequent reaction to yield sulfite [52,53].

To produce sulfide for amino acid biosynthesis, sulfite must undergo further reduction through sulfite reductase [53]. However, a gene encoding sulfite reductase could not be identified, suggesting that *Hrs. convoluta* may employ an unusual reductase or an alternative mechanism to perform this reaction. Genes encoding all successive enzymes necessary to synthesize cysteine, homocysteine, and methionine from hydrogen sulfide were identified (data not shown). By comparison, the *Hbt. modesticaldum* genome lacked *cysN*, *cysH*, and sulfite reductase, supporting physiological studies indicating that *Hbt. modesticaldum* requires a reduced sulfur source for biosynthetic purposes [16].

Interestingly, cultures of *Hrs. convoluta* strain HH^T^ were able to grow well in the presence of high levels of sulfide (10mM), with sulfide oxidation accompanied by the production of elemental sulfur globules during growth [21]. However, the pathway for this reaction remains unclear, as the *Hrs. convoluta* genome appears to lack genes encoding traditional sulfide oxidoreductases, such as the sulfide:quinone oxidoreductase (SQR) from the green sulfur bacterium *Chlorobaculum* (*Chlorobium) tepidum* that oxidizes H_2_S to S^0^ and reduces quinone [55], or sulfide:flavocytochrome *c* oxidoreductase from the purple sulfur bacterium *Allochromatium vinosum* that oxidizes sulfide to sulfur or polysulfides [56]. Thus, it is possible that *Hrs. convoluta* contains a novel sulfide oxidoreductase for this purpose.

### 3.5. Photosynthesis Genes and Pigment Biosynthesis

**Figure 5:**
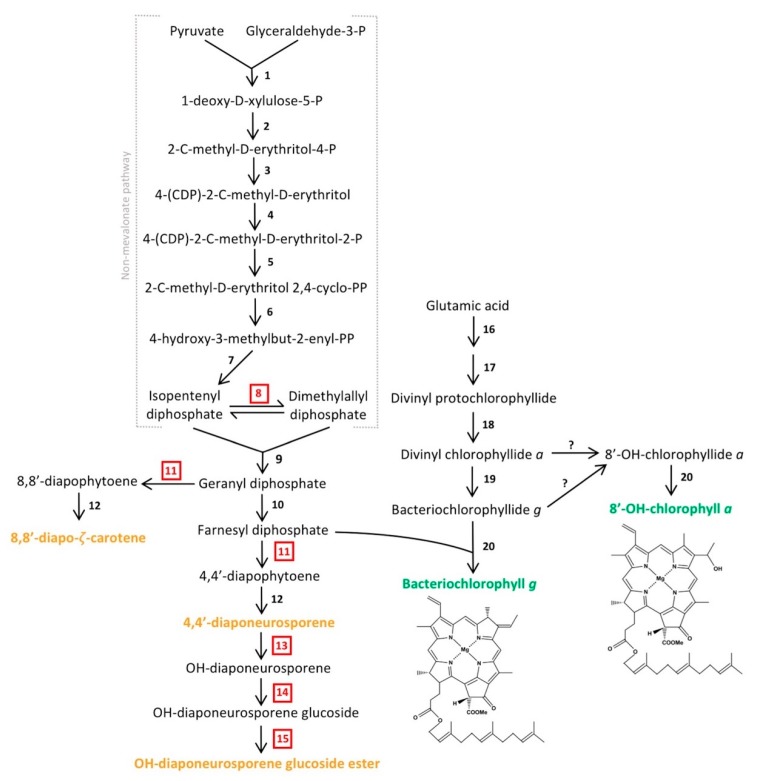
Predicted biosynthetic pathway of major pigments in *Heliorestis convoluta*. The non-mevalonate pathway shows the synthesis of farnesyl diphosphate for either conversion into carotenoids (orange) or incorporation into the final chlorophyll (green) structures. The enzymes that catalyze each individual numbered reaction are (1) 1-deoxy-d-xylulose-5-P synthase, (2) 1-deoxy-d-xylulose-5-P reductoisomerase, (3) 4-(CDP)-2-C-methyl-d-erythritol synthase, (4) 4-(CDP)-2-C-methyl-d-erythritol kinase, (5) 2-C-methyl-d-erythritol 2,4-cyclo-PP synthase, (6) 4-hydroxy-3-methylbut-2-enyl-PP synthase, (7) 4-hydroxy-3-methylbut-2-enyl-PP reductase, (8) isomerase, (9) geranyl diphosphate synthase, (10) farnesyl diphosphate synthase, (11) 4,4′-diapophytoene synthase, (12) diapophytoene dehydrogenase, (13) hydratase, (14) glucosyl transferase, (15) esterase, (16) enzymes encoded by *gltx* and *hemALBCDENYG* genes, (17) enzymes encoded by *bchIDHME* genes, (18) protochlorophyllide reductase (*bchLNB*), (19) chlorophyllide reductase (*bchXYZ*), and (20) bacteriochlorophyll synthase (*bchG*). Red, boxed numbers represent enzymes not yet identified in the *Hrs. convoluta* genome but are proposed based on the predicted pathway. Adapted from Takaichi et al. [63], *Arch. Microbiol.* **2003**, *179*, 95–100. Copyright 2002 Springer Nature; Dubey et al. [64] *J. Biosci.* **2003**, *28*, 637–646. Copyright 2003 Springer Nature; Sattley et al. [6] *J. Bacteriol.* **2008**, *190*, 4687–4696. Copyright 2008 American Society for Microbiology; Sattley and Swingley [7], *Adv. Bot. Res.* **2013**, *66*, 67–97, Copyright 2013 Elsevier Ltd.; and Tsukatani et al. [60], *Biochim. Biophys. Acta* **2013**, *1827*, 1200–1204. Copyright 2013 Elsevier Ltd.

Heliobacteria synthesize bacteriochlorophyll (BChl) *g*, a pigment absorbing light maximally between 785 and 790 nm, for phototrophic growth [4]. Accordingly, genes encoding enzymes that catalyze the conversion of glutamic acid to divinyl protochlorophyllide (*gltX*, *hemALBCDEN,* and *bchIDHME*) for pigment biosynthesis (Figure 5) were annotated in *Hrs. convoluta*. However, as for *Hbt. modesticaldum*, neither of the genes encoding protoporphyrinogen oxidase (*hemY* or *hemG*), which catalyzes the oxidation of protoporphyrinogen to protoporphyrin, was identified in the *Hrs. convoluta* genome. Moreover, comparisons with *hemG* from *Escherichia coli* and *hemY* from *Bacillus subtilis* yielded no significant sequence identity to genes in the *Hrs. convoluta* genome. Due to the anaerobic nature of *Hrs. convoluta*, an alternative and unidentified enzyme likely acts as a dehydrogenase rather than an oxidase in this step of pigment biosynthesis. Studies with *Desulfovibrio gigas*, also a strict anaerobe, suggest that electron carriers, such as flavins and pyridine nucleotides, or electron-transport complexes, such as nitrite and fumarate reductases, do not use O_2_ as the electron acceptor in the conversion of protoporphyrinogen to protoporphyrin [57,58]. More recently, however, an alternative pathway that does not use protoporphyrin to synthesize heme has been described in *Hbt. modesticaldum* [59], and a similar mechanism likely exists in *Hrs. convoluta*. 

Following the synthesis of divinyl protochlorophyllide in *Hrs. convoluta*, genes encoding protochlorophyllide reductase (*bchLNB*), chlorophyllide reductase (*bchXYZ*), and bacteriochlorophyll synthase (*bchG*) are present to facilitate catalysis of subsequent reactions and produce BChl *g*. Previous work with *Hbt. modesticaldum* suggested the need for an isomerase in the interconversion between 8-vinyl bacteriochlorophyllide *a* and bacteriochlorophyllide *g* [6], but more recent experimental work with this species revealed the ability of chlorophyllide reductase to perform both reduction and isomerization of divinyl chlorophyllide *a* and circumvent the need for a separate isomerase in the biosynthesis of bacteriochlorophyllide *g* [60,61].

Heliobacteria also contain an alternative form of chlorophyll (Chl) *a*, 8^1^-OH-Chl *a*, which was observed as a smaller absorption peak at 672 nm in spectrophotometric studies of *Hrs. convoluta* [21]. Whereas BChl *g,* a bacteriochlorin-type chlorophyll, is reduced at the C-7 and C-8 bond and has an ethylidene functional group at C-8 [5], 8^1^-OH-Chl *a*, a chlorin, has a double bond connecting C-7 and C-8 with a hydroxyethyl group at C-8 [44]. BChl *g* and 8^1^-OH-Chl *a* are putatively synthesized from a common precursor, divinyl chlorophyllide *a* [7,60].

Hydration of the C-8 vinyl group of divinyl chlorophyllide *a* is catalyzed by 8-vinyl chlorophyllide hydratase, and bacteriochlorophyll synthase catalyzes the addition of a farnesyl group to produce the mature 8^1^-OH-Chl *a* [7,60]. However, a gene encoding chlorophyllide hydratase or an analogous enzyme was not identified in the genomes of *Hrs. convoluta* or *Hbt. modesticaldum* [6]. Hence, a possible alternative mechanism for 8^1^-OH-Chl *a* synthesis includes steps of dehydrogenation and subsequent hydroxygenation of bacteriochlorophyllide *g* to produce 8^1^-OH-chlorophyllide *a* [60], but genes encoding enzymes for this reaction were not identified in either *Hrs. convoluta* or *Hbt. modesticaldum*. Yet another possible mechanism for 8^1^-OH-Chl *a* synthesis would require the irreversible conversion of BChl *g* into 8^1^-OH-Chl *a* upon exposure to O_2_ and light [62]. However, as strict anaerobes, the viability of heliobacteria is compromised upon exposure to O_2_, and therefore this mechanism is unlikely as the major pathway for 8^1^-OH-Chl *a* production [3,62].

**Figure 6:**
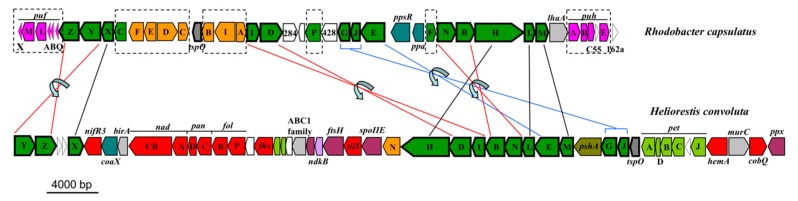
Photosynthesis gene clusters from *Heliorestis convoluta* and the purple bacterium *Rhodobacter capsulatus*. Shared genes are outlined in bold. Lines indicate gene synteny: black, single gene rearrangements; red, inverted genes; and blue, inverted genes with a gene insertion. Dashed boxes show *Rba. capsulatus* photosynthesis genes absent from *Hrs. convoluta*. Colors: green, bacteriochlorophyll biosynthesis (bch); orange, carotenoid biosynthesis (crt); pink, proteobacterial reaction centers (puf) and light harvesting complexes (puh); olive, heliobacterial reaction center (psh); teal, regulatory proteins; light green, electron transport (pet); red, cofactor biosynthesis; purple, cell division and sporulation; light blue, nitrogen fixation; grey, transcription; light grey, other nonphotosynthesis genes; and white, uncharacterized genes. Adapted from Sattley et al. [6]. *J. Bacteriol.* **2008**, *190*, 4687–4696. Copyright 2008 American Society for Microbiology.

Due to high sequence identity between genes allowing for phototrophic growth (data not shown), a mechanism similar to BChl *g* and 8^1^-OH-Chl *a* biosynthesis in *Hbt. modesticaldum* [6,7,60] is predicted for *Hrs. convoluta* (Figure 5). Many of the genes encoding enzymes required for pigment biosynthesis are grouped into a single photosynthesis gene cluster (PGC) in heliobacteria. The PGCs of *Hrs. convoluta* and *Hbt. modesticaldum* were nearly identical and displayed a shared gene synteny in all key genes, including those associated with pigment and cofactor biosynthesis, electron transport, and light harvesting, suggesting that a common genetic architecture—one that differs substantially from the PGCs present in the genomes of purple bacteria—defines the heliobacterial PGC (Figure 6).

Like BChls *c*, *d,* and *e* of green sulfur bacteria, both BChl *g* and 8^1^-OH-Chl *a* of heliobacteria are esterified with farnesol [63]. A non-mevalonate pathway is employed to synthesize the esterifying alcohol, farnesyl diphosphate, of heliobacterial pigments [64]. As was noted for *Hbt. modesticaldum*, *Hrs. convoluta* contained the complete complement of genes for this pathway, beginning with pyruvate and glyceraldehyde-3-phosphate and proceeding to an unidentified but predicted isomerase that could catalyze the interconversion of isopentenyl diphosphate and dimethylallyl diphosphate [6,64] (Figure 5). Following this, farnesyl diphosphate can either be incorporated into the final structures of BChl *g* and 8^1^-OH-Chl *a* or further transformed into the major carotenoids found in *Hrs. convoluta* [63] (Figure 5). The high specificity of BchG for incorporation of a farnesol moiety over longer alcohol groups, such as phytol, has been demonstrated in studies of pigment biosynthesis in *Hbt. modesticaldum* [61], and the high sequence identity (68%) of BchG from *Hrs. convoluta* to that of *Hbt. modesticaldum* suggests a similar activity in the alkaliphile.

Experimental work and pigment extraction from *Hrs. convoluta*, *Hrs. daurensis*, and *Hrs. baculata* revealed that the major carotenoid in alkaliphilic heliobacteria is OH-diaponeurosporene glucoside C16:0 ester, followed by 4,4′-diaponeurosporene, OH-diaponeurosporene glucoside C16:1 ester, and 8,8′-zeta-carotene [21,63]. These novel glucoside esters in alkaliphilic heliobacteria were not found in neutrophilic heliobacteria, in which 4,4′-diaponeurosporene was the major carotenoid [63,65]. The synthesis of these C_30_ carotenoids [66] is complicated by the apparent absence of a gene (*crtM*) encoding 4,4′-diapophytoene synthase in both *Hbt. modesticaldum* [6] and *Hrs. convoluta*. Presumably, the presence of an enzyme with 4,4′-diapophytoene synthase activity is essential in the proposed biosynthetic pathway for each of the carotenoids found in heliobacteria [63] (Figure 5). Although *crtM* was not identified, two nonidentical copies of *crtN* (FTV88_2648 and FTV88_3059) having a sequence identity of 71% were annotated in the *Hrs. convoluta* genome, and it is possible that one of their gene products exhibits CrtM-like activity.

In alkaliphilic heliobacteria, a proposed CrtC-like hydratase catalyzes the formation of OH-diaponeurosporene from 4,4′-diaponeurosporene, followed by synthesis of OH-diaponeurosporene glucoside by a CrtX-like glucosyl transferase, with a putative esterase making the final conversion to the mature glucoside ester [63]. Genes encoding the enzymes catalyzing the final three steps of OH-diaponeurosporene glucoside ester synthesis were not identified in *Hrs. convoluta* (Figure 5), but genes encoding two carotenoid biosynthesis proteins (FTV88_0301 and FTV88_0302) were annotated. These genes showed no significant sequence similarity to genes of the neutrophilic *Hbt. modesticaldum*, and they may be candidates for encoding proteins to perform the final steps of carotenoid biosynthesis in alkaliphilic heliobacteria.

### 3.6. Reaction Center and Electron Transport Chain

Heliobacteria possess a type I (Fe–S type) photosynthetic reaction center (RC) imbedded in the cytoplasmic membrane [4,67,68]. As the simplest known and perhaps most ancient extant (bacterio)chlorophyll-binding photochemical apparatus [69], the heliobacterial RC is a symmetrical homodimer consisting of the PshA polypeptide and the novel, single-transmembrane helix PshX polypeptide [70]. PshA of *Hrs. convoluta* (encoded by *pshA*, FTV88_2638) showed 71% sequence identity to PshA of *Hbt. modesticaldum* but nearly 96% identity to PshA of *Hrs. acidaminivorans* (GenBank accession WBXO01000000, unpublished). As is the case in *Hbt. modesticaldum*, *pshX* (FTV88_2551) is situated outside of the PGC in *Hrs. convoluta* and encodes a protein consisting of just 31 amino acids. The PshX RC subunit from *Hrs. convoluta* showed a 74% sequence identity to that of *Hbt. modesticaldum*.

The crystal structure of the *Hbt. modesticaldum* RC revealed the presence of 54 BChl *g* molecules, two 8^1^-OH-Chl *a* molecules, two carotenoids (4,4′-diaponeurosporene), four BChl *g*′ molecules (a C-13 epimer of BChl *g* that functions as the primary electron donor, P_800_) [68,71,72], two lipids, and one [4Fe–4S] cluster [70]. Experimental data on the structure of the *Hrs. convoluta* RC are not yet available. However, with their highly similar PshA and PshX proteins, the geometry and pigment composition of the *Hrs. convoluta* RC should closely resemble that of the *Hbt. modesticaldum* RC [69]. Nevertheless, some distinctions may materialize considering the alternative carotenoids produced by alkaliphilic heliobacteria and their inherently alkaline habitat [63].

**Figure 7:**
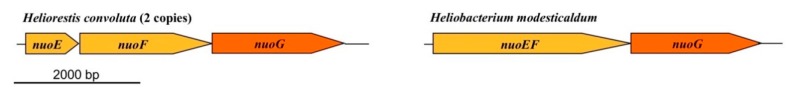
Comparison of *nuoEFG* genes in *Heliorestis convoluta* and *Heliobacterium modesticaldum*. Whereas in *Hbt. modesticaldum* (and *Heliobacillus mobilis*) *nuoE* and *nuoF* are fused, these genes are independent in *Hrs. convoluta*. In both species, however, *nuoEFG* are separated from other *nuo* genes on the chromosome. In addition, unlike in *Hbt. modesticaldum*, *Hrs. convoluta* contains two copies of *nuoEFG*, as well as the *hydEFG* maturase genes (FTV88_1003–1005) that may impart [FeFe] hydrogenase activity. Colors: gold, NADH dehydrogenase subunits; orange, structural genes.

Proteins of the electron transport chain (ETC) of *Hrs. convoluta* exhibited high sequence similarity to those from *Hbt. modesticaldum*, and thus the overarching mechanism of light-driven energy conservation is likely to be highly conserved across all heliobacterial taxa. Although not experimentally confirmed, it is likely that electrons first enter the chain by the activity of either NADH:quinone oxidoreductase (Figure 3), a 14-subunit protein complex embedded in the cytoplasmic membrane and encoded by *nuoABCDEFGHIJKLMN*, or perhaps a complex having ferredoxin:menaquinone oxidoreductase activity. As observed in *Hbt. modesticaldum*, the *nuoEFG* genes in *Hrs. convoluta* are not co-localized within the same operon as the other *nuo* genes. However, unlike in *Hbt. modesticaldum*, in which *nuoEF* are fused, *nuoE* and *nuoF* exist as individual genes (present in duplicate copies) in *Hrs. convoluta* (Figure 7). With the exception of this distinction, all *nuo* genes show high sequence identity (62–79%) between the two species. As for *Hbt. modesticaldum*, menaquinone is predicted to shuttle electrons from Complex I to the cytochrome *bc* complex (PetABCD), and electron transfer through these complexes drives translocation of H^+^ to the periplasmic space (Figure 3), forming a proton motive force (PMF) [6,73].

Cytochrome *bc*_1_ complexes, which are found in a variety of anoxygenic phototrophs and also in eukaryotic mitochondria, consist of a minimum of three protein subunits: cytochrome *b*, cytochrome *c*_1_, and the Rieske iron-sulfur protein [74]. In contrast, the related cytochrome *b*_6_*f* complex, which is present in cyanobacteria and chloroplasts, is comprised of cytochrome *f* (PetA), cytochrome *b*_6_ (PetB), the Rieske iron-sulfur protein (PetC), and subunit IV (PetD) [74]. Having similar functions but distinct structural properties, cytochrome *b* contains eight transmembrane helices, whereas cytochrome *b*_6_ and its associated subunit IV contain four and three transmembrane helices, respectively [74]. Cytochrome *b*_6_ shows homology to the *N*-terminal half of cytochrome *b*, and subunit IV is homologous to the *C*-terminal half of cytochrome *b* [74].

An analysis of the cytochrome *bc* complex of *Hrs. convoluta* indicated that it resembles a hybrid of the cytochrome *b*_6_*f* complex and the cytochrome *bc*_1_ complex. A comparison of cytochrome *b*_6_ and subunit IV proteins from *Hrs. convoluta* and the model cyanobacterium *Synechocystis* PCC 6803 showed 48% and 42% amino acid sequence identity, respectively. However, cytochrome *b*_6_ and subunit IV from *Hrs. convoluta* also showed 36% and 30% amino acid identity, respectively, to the *N*-terminal and *C*-terminal halves of cytochrome *b* from the purple bacterium *Rhodobacter sphaeroides*. Furthermore, whereas subunit IV from *Hrs. convoluta* is predicted to contain the usual three transmembrane helices, cytochrome *b*_6_ from *Hrs. convoluta* contained a predicted five transmembrane regions instead of the four typically observed in the *b*_6_*f* complex. Notably, cytochrome *b*_6_ from *Hbt. modesticaldum* is predicted to contain the conventional four transmembrane helices. Therefore, considering the above sequence analyses and their total of eight predicted transmembrane helices, the PetB and PetD proteins of *Hrs. convoluta* may represent a structural and evolutionary intermediate between cytochrome *b* and cytochrome *b*_6_/subunit IV proteins, a distinction perhaps not shared with neutrophilic heliobacteria.

The PetA protein in heliobacteria is also of interest because it functions as a diheme cytochrome *c* (as opposed to the typical monoheme protein) and shows no sequence or structural similarity to cytochrome *f* [74]. Although unusual among the *Firmicutes*, the diheme cytochrome *c* has been identified in all heliobacteria studied thus far and is likely a universal feature of these phototrophs. PetA from *Hrs. convoluta* showed high sequence identity with PetA from *Hrs. acidaminivorans* (79%), and sequence identities to PetA from neutrophilic heliobacteria (e.g., *Hbt. modesticaldum*, *Hbt. gestii*, *Heliobacillus mobilis*, and *Heliophilum fasciatum*) were all near 50%. Based on similarities in its N- and C-terminal domains, the heliobacterial diheme cytochrome *c* may have been the result of a past gene duplication and subsequent fusion [75]. 

A single operon containing all eight genes encoding the subunits of ATP synthase (*atpABCDEFGH*) was identified in the genome of *Hbt. modesticaldum* [6,26], and the encoded ATP synthase itself has since been biochemically characterized [76]. The composition and arrangement of ATP synthase genes in *Hrs. convoluta* was identical to that in the *Hbt. modesticaldum* genome. Kinetic studies with *Hba. mobilis* and *Hbt. modesticaldum* and physiological similarity to photosystem I of cyanobacteria suggest that a PMF established by cyclic electron flow drives photophosphorylation in heliobacteria [73,77,78]. For overviews of electron transfer reactions in heliobacteria, see Sattley and Swingley [7], Kondo et al. [79], and, more recently, Kashey et al. [73].

### 3.7. Endosporulation

A likely universal trait of heliobacteria is the ability to form endospores [11], differentiated and largely dormant cells that are highly resistant to environmental extremes, such as heat and desiccation. Genomic comparisons of *Hrs. convoluta* and *Hbt. modesticaldum* revealed high similarity between endosporulation genes in each species. For example, genes encoding key sporulation sigma factors (σ^H^, σ^E^, σ^F^, σ^G^, σ^K^) in *Hbt. modesticaldum* were also identified in the *Hrs. convoluta* genome. Like *Hbt. modesticaldum*, *Hrs. convoluta* lacked the *spo0M* gene functioning to regulate stage 0 development of endosporulation [80] and the *spoIIB* gene necessary for robust sporulation in *B. subtilis* [81]. This may help explain the sporadic (as opposed to consistent) production of endospores in serially subcultured cells of *Hrs. convoluta* strain HH^T^ [21], as the deletion of either *spo0M* or *spoIIB* in *B. subtilis* results in impairment of endosporulation [80,81]. Additionally, the 20 *cot* genes encoding proteins that comprise the protective spore coat for *B. subtilis*, including *cotH* required for spore coat assembly [82], did not show significant similarity to genes in *Hbt. modesticaldum* [6] or *Hrs. convoluta*. Likewise, key proteins that coordinate spore coat assembly and composition in *Clostridioides* (*Clostridium*) *difficile*, including CotA and CotB [83], showed no sequence similarity to genes in *Hrs. convoluta*. Despite these deficiencies, cells of *Hrs. convoluta* strain HH^T^ were still capable of forming heat-resistant endospores, even if sporadically [21]. These findings suggest shared biosynthetic and regulatory mechanisms governing endosporulation in *Hbt. modesticaldum* and *Hrs. convoluta* that differ in some respects from those that govern endosporulation in species of *Bacillus* and *Clostridium*.

### 3.8. Molecular Adaptations to Alkaliphily in *Heliorestis convoluta*

Alkaliphilic bacteria employ several mechanisms to maintain intracellular pH homeostasis in their highly alkaline environments. Experimental work conducted with alkaliphiles revealed that these organisms maintain a lower cytoplasmic pH than their external environment—up to a 2.3 pH unit difference—for optimal enzyme activity and cellular functioning [84,85]. Despite its optimal growth pH of 8.5–9 and ability to grow slowly at pH 10 [21], it is likely that *Hrs. convoluta* maintains a cytoplasmic pH at or below pH 8, as is true from studies of several alkaliphilic strains of *Bacillus* [84,86].

Cytoplasmic pH homeostasis in *Hrs. convoluta* is likely supported by the presence of a Na^+^/H^+^ antiporter encoded by *nhaA* (FTV88_0116). To maintain cytoplasmic pH at homeostatic levels, the Na^+^/H^+^ antiporter operates in an electrogenic manner, facilitating the import of twice as many H^+^ as Na^+^ exported [87,88]. The inward movement of protons through the antiporter acidifies the cytoplasm to maintain a pH closer to neutral [87,88,89]. The NhaA protein from *Hrs. convoluta* was found to be 87% identical in amino acid sequence to NhaA from *Heliorestis acidaminivorans*—also an alkaliphile—but only 50% identical to NhaA from the neutrophile *Hbt. modesticaldum*. The NhaA enzyme may, therefore, be a good candidate to study which amino acid residues facilitate antiporter activity in alkaline versus neutral environments.

In addition to its role in cytoplasmic pH maintenance, the Na^+^/H^+^ antiporter generates a sodium motive force (SMF) that has been shown to be important for secondary active transport of various substrates [89,90,91] (see Figure 3 for examples in *Hrs. convoluta*). The use of Na^+^-coupling for transport is potentially more important in *Hrs. convoluta* than in its neutrophilic relative, *Hbt. modesticaldum*, as genes encoding multiple Na^+^-dependent transporters (FTV88_2418 and FTV88_1400) and a Na^+^/Ca^+^ antiporter (FTV88_2739) in *Hrs. convoluta* showed little to no significant similarity with genes in *Hbt. modesticaldum*. 

The more neutral cytoplasm compared to the alkaline extracellular milieu would seemingly create an outward-directed bulk PMF rather than the inward-directed PMF needed to drive ATP synthesis [89,90,91]. Despite this, most alkaliphiles, including *Hrs. convoluta*, still employ a PMF rather than a SMF to power ATP synthase [89,92]. Alkaliphilic bacteria must therefore have mechanisms in place to prevent H^+^ equilibration with the external environment so that an effective local PMF can be established. To this end, carotenoids, which are produced in large quantities by alkaliphilic heliobacteria [93], have been proposed to play a role in organizing proton pumps close to ATP synthases in the membrane, thus facilitating more efficient ATP generation [91,94,95]. In addition, cardiolipin, a glycerophospholipid that assists in membrane domain organization, may also help prevent H^+^ equilibration by functioning as a proton sink for the H^+^-coupled ATP synthase [96,97,98]. By functioning in this capacity, cardiolipin allows for the retention of H^+^ near the surface of the cell membrane so that they are unable to spontaneously diffuse into the alkaline environment. Notably, a gene encoding cardiolipin synthase was present in *Hrs. convoluta* (FTV88_2523), but no corresponding homolog was identified in *Hbt. modesticaldum*.

**Figure 8:**
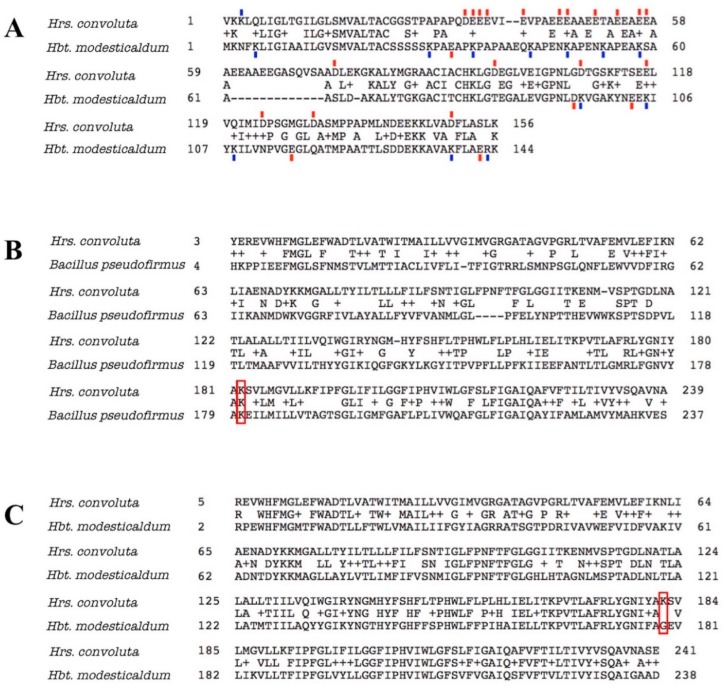
Amino acid sequence alignments for cytochrome *c*_553_ and ATP synthase F_o_ alpha subunit of *Heliorestis convoluta* with related species. (**A**) The sequence alignment for cytochrome *c*_553_ of *Hrs. convoluta* and *Heliobacterium modesticaldum*. Acidic amino acid residues (red), aspartate (D) and glutamate (E), and basic amino acid residues (blue), arginine (R) and lysine (K), that differed between each species were indicated by a colored dash directly above or below the residue. Acidic or basic amino acids in the gap (–) regions were not marked. (**B**) Sequence alignment for ATP synthase F_o_ alpha subunit of *Hrs. convoluta* and *Bacillus pseudofirmus* OF4. The lysine residue of interest at position 180 in *B. pseudofirmus* aligns with Lys^182^ in *Hrs. convoluta* (red box). (**C**) Sequence alignment for ATP synthase F_o_ alpha subunit of *Hrs. convoluta* and *Hbt. modesticaldum*. The lysine residue of interest at position 182 in *Hrs. convoluta* aligns with Gly^179^ in *Hbt. modesticaldum* (red box). All sequence alignments were generated using the BLAST algorithm.

In addition to producing proteins and other molecules that counteract the pH difference between the cytoplasm and environment and the consequences thereof, homologous proteins also have amino acid substitutions that optimize the functioning of normal processes for the alkaline environment. In alkaliphiles, the portions of extracellular enzymes that are exposed to the external environment tend to have decreased numbers of basic residues (arginine, histidine, or lysine), with acidic amino acids (aspartate or glutamate) or neutral residues in their place [99,100,101]. In a noteworthy example, the amino acid sequence for cytochrome *c*_553_ (PetJ) of *Hrs. convoluta* contained 13 more acidic amino acid residues and 11 fewer basic residues than PetJ of *Hbt. modesticaldum* (Figure 8A). In line with previous discussion, the elevated number of acidic residues and corresponding decrease in basic residues in the externally-functioning *Hrs. convoluta* PetJ should contribute to OH^−^ repulsion and H^+^ attraction near the membrane surface and help maintain the PMF [89]. Although additional investigation of the cell surface of *Hrs. convoluta* is required to confirm its electrochemical nature, genomic data suggest that this phototroph can sequester H^+^ near the cell surface to create an effective PMF for ATP synthesis and flagellar motility.

In a similar way, several key amino acid residues and motifs in ATP synthase have been found to contribute to optimal functioning of the enzyme at different pH levels [89,91,102,103]. For example, a lysine residue found at position 180 in the F_o_ alpha subunit of ATP synthase in *Bacillus pseudofirmus* OF4 was determined to favor H^+^-powered ATP synthesis at an alkaliphilic pH due to its basic properties [102,104,105,106]. As expected, a corresponding Lys^182^ in the *Hrs. convoluta* F_o_ alpha subunit (Figure 8B) can presumably capture protons optimally from the alkaline environment and release them into the rotor subunit of ATP synthase at an external basic pH near the high pKa of the side chain [102]. The lysine residue would be detrimental to ATP synthesis in a neutral pH range, as H^+^ would be retained on the residue side chain at ~pH 7 (below the side chain pKa). This highlights the significance of a glycine residue at the corresponding position in the F_o_ alpha subunit in neutrophilic bacteria, including *Hbt. modesticaldum* [102] (Figure 8C).

Several alkaliphilic bacteria use a SMF to power flagellar motor proteins, thus reserving the valuable and limited PMF for ATP production [107,108]. Research conducted with alkaliphilic *Bacillus* spp. concluded that a highly conserved valine residue is present in H^+^-driven (MotB) flagellar motor protein sequences, whereas a leucine residue takes the place of this valine in Na^+^-driven (MotS) motor protein sequences [107,108]. The alkaliphilic *Bacillus* spp. contained MotS with the conserved leucine amino acid, allowing these bacteria to use a SMF to power Na^+^-coupled flagellar motility [107,108]. Interestingly, MotB—with its conserved valine—was identified in both *Hrs. convoluta* and *Hbt. modesticaldum*, suggesting that a PMF is used to power motility in both alkaliphilic and neutrophilic heliobacteria. Genomic analyses confirmed the presence of a core set of 24 genes (*fliCDEFGHIMNPQR*, *flgBCDEFGKL*, *motAB*, *flhAB*) in *Hrs. convoluta* that are essential for PMF-driven swimming motility in numerous flagellated bacteria [109].

## 4. Conclusions

The analysis of the complete genome sequence of *Hrs. convoluta* has provided further insight into the photoheterotrophic metabolism, nitrogen utilization, sulfur assimilation, and pigment biosynthesis pathways of heliobacteria, as well as molecular adaptations to an alkaliphilic existence. Further biochemical and genetic experimentation with alkaliphilic heliobacteria, including *Hrs. convoluta*, is necessary to confirm genomics-based predictions regarding the roles of specific genes and the apparent absence of specific enzyme activities.

## Figures and Tables

**Table 1:** Comparison of genome features of *Heliorestis convoluta* str. HH^T^ and *Heliobacterium modesticaldum* str. Ice1^T^ [6].

Characteristic	*Hrs. convoluta*	*Hbt. modesticaldum*
Chromosome size (bp)	3,218,981	3,075,407
G + C content (%)	43.1	56.0
Coding DNA (%)	86.9	87
Protein-encoding genes (no.)	3,263	3,138
Average gene length (bp)	855	882
ATG initiation codons (%)	63.5	62.1
GTG initiation codons (%)	15.7	19.1
TTG initiation codons (%)	20.8	18.8
rRNAs (no.)	9	24
tRNAs (no.)	105	104
Transposases (no.)	18	70
Putative pseudogenes (no.)	22	8
CRISPR repeats (no.)	1	Not determined

**Table 2:** Functional role categories of *Heliorestis convoluta* str. HH^T^ genes.

Characteristic	Genes	% of Genome Content *
Amino acid biosynthesis	119	3.64
Biosynthesis of cofactors, prosthetic groups, and carriers	142	4.35
Cell envelope and surface features	216	6.61
Cellular processes (cell division, motility, sporulation, etc.)	477	14.6
DNA metabolism	225	6.88
Energy and central intermediary metabolism	512	15.94
Fatty acid and phospholipid metabolism	66	2.02
Mobile and extrachromosomal element functions	76	2.33
Protein synthesis and fate	338	10.34
Purines, pyrimidines, nucleosides, and nucleotides	59	1.81
Regulatory functions	137	4.19
Signal transduction	80	2.45
Transcription	142	4.35
Transport and binding proteins	342	10.47
Hypothetical proteins	899	27.51

* Total exceeds 100%, as some genes are assigned to more than one role category.
